# The Effects of a VR Teaching Tool on Understanding, Comfortability, Compassion, and Empathy of Students and Caregivers

**DOI:** 10.1093/geroni/igab046.233

**Published:** 2021-12-17

**Authors:** Carrie Elzie

**Affiliations:** Eastern Virginia Medical School, Norfolk, Virginia, United States

## Abstract

Empathetic care giving is associated with improved patient satisfaction, compliance and outcomes; clinical competence, career satisfaction, and burnout reduction; as well as diminished medical errors and litigation claims. Unfortunately, recent studies have shown erosion in empathy and compassion across the health professions. Virtual reality shows promise as a teaching tool to combat this decline as it has been dubbed the ultimate empathy machine, allowing users to vividly and viscerally experience any situation from any perspective. Embodied Labs allows users to virtually walk in the shoes of different patients, experiencing symptoms, family dynamics, support networks and various components of the health care systems. We have demonstrated that the high level of immersion and presence afforded by these virtual labs are effective pedagogical tools to increase understanding, comfortability, compassion and empathy within various populations including informal caregivers, high school students, health professional students and medical students.

